# Molecular Insights of Nickel Binding to Therapeutic Antibodies as a Possible New Antibody Superantigen

**DOI:** 10.3389/fimmu.2021.676048

**Published:** 2021-07-08

**Authors:** Chinh Tran-To Su, Wai-Heng Lua, Jun-Jie Poh, Wei-Li Ling, Joshua Yi Yeo, Samuel Ken-En Gan

**Affiliations:** ^1^ Antibody & Product Development Lab, Experimental Drug Development Centre, Bioinformatics Institute, Agency for Science, Technology, and Research (A*STAR), Singapore, Singapore; ^2^ James Cook University, Singapore, Singapore; ^3^ APD SKEG Pte Ltd, Singapore, Singapore

**Keywords:** allergy, glutamine, type I hypersensitivity, IgG1, IgE, nickel (II), antibody

## Abstract

The binding of nickel by immune proteins can manifest as Type IV contact dermatitis (Ni-specific T cells mediated) and less frequently as Type I hypersensitivity with both mechanisms remaining unknown to date. Since there are reports of patients co-manifesting the two hypersensitivities, a common mechanism may underlie both the TCR and IgE nickel binding. Focusing on Trastuzumab and Pertuzumab IgE variants as serendipitous investigation models, we found Ni-NTA interactions independent of Her2 binding to be due to glutamine stretches. These stretches are both Ni-inducible and in fixed pockets at the antibody complementarity-determining regions (CDRs) and framework regions (FWRs) of both the antibody heavy and light chains with influence from the heavy chain constant region. Comparisons with TCRs structures revealed similar interactions, demonstrating the possible underlying mechanism in selecting for Ni-binding IgEs and TCRs respectively. With the elucidation of the interaction, future therapeutic antibodies could also be sagaciously engineered to utilize such nickel binding for biotechnological purposes.

## Introduction

Nickel (Ni) is ubiquitous in household products and food (1), and can cause allergic reactions for ~15% of the population (2) making it one of the most common metal allergies (3). It can manifest as either type IV contact dermatitis mediated by hapten-specific T cells (2) and very occasionally as IgE-mediated type I hypersensitivity (4). Associations between the two allergies are increasingly reported (5, 6) where they can occur within the same patient (4). The similarities in the structural topologies between the antibody variable (V) regions (complementarity-determining regions or CDRs and framework regions or FWRs) with those of T cell receptor (TCR) may provide a clue to a possible common pathogenesis (7–9) from the clonal selection and expansion of such Ni reacting T- and B-cells. Known to be shuttled by albumin and other nickel binding proteins (10), metal-protein complexes formed with nickel can activate T-cells and also cross-link nickel-specific IgEs (11). Thus, the elucidation of the mechanism in one immune protein (e.g. IgE) may also provide insight into TCR activation which can in itself play the dual role in selectively expanding type IV and type I hypersensitivity effector cells, where activated CD4+ cells can also support the IgE producing B-cells *via* linked recognition (12).

Histidine binding of Ni^2+^ ions is long-known and has been exploited in his-tag protein affinity purification (13). This also underlies the interaction of the major histocompatibility complex (MHC) with certain peptides (14, 15), Hpn-like protein (16), in Fcγ (17), and in TCRs of type IV contact dermatitis patients (18). In biologics, this mechanism may partly account for the dermatological adverse effects associated with certain targeted therapies (19–21), where apart from directly causing type I and IV responses, the metal binding can also contribute to protein aggregation (22), increasing immunogenicity (23).

Ni IgE-mediated hypersensitivity occurs most commonly as occupational hazards (24), where the prolonged exposures elicited Ni-interacting IgEs (25) through possible “non-antigenic” mechanisms (26) that arise from the expansion of existing IgEs (capable of additionally binding Ni) producing B-cells. We hypothesize the Ni-only specific IgEs to be extremely rare given the chemical nature of Ni, but rather that the unintended non-specific binding to Ni during antibody repertoire generation to other antigens occurred and were gradually selected for and expanded. To investigate this possible mechanism, a holistic investigation (27) of whole IgEs to study the various antibody regions and their possible combination to structurally form histidine-rich Ni-binding patches are required. This likelihood has support from previous findings showing that different antibody regions interact synergistically in antigen binding (28, 29) and FcR engagement (30, 31).

While superantigens are classically defined to directly bind and activate TCRs (32), their classifications have been expanded to include microbial proteins that bind to immunoglobulins, termed B-cell (33) or Ig/antibody superantigens e.g. Proteins A and L (34). These antibody superantigens can bind specifically to antibody regions without being recognized as conventional antigens and are often used for purification. Despite having T-cell superantigen-like properties, nickel was excluded as a superantigen on the basis of requiring specific interactions as with conventional TCR superantigens (35). Nonetheless, this exclusion may be challenged when applied to antibody superantigens where the known protein L superantigen interaction with antibody FWR1 can be affected by deletions in FWR3 while largely keeping the equivalent VH-VL pairing (36).

Nonetheless, since the binding of both TCR- and antibodies can arise from a common superantigen-like mechanism, an in-depth molecular investigation is warranted.

Fortuitously, our recombinant Trastuzumab and Pertuzumab IgE VH-FWR swapped variants (30) were found to bind Ni-NTA without diminishing antigen (Her2) specificity. It is from this panel that we sought to unravel the binding mechanism of Ni to the IgEs.

## Materials and Methods

### Cell Culture

HEK EXPI293F (GIBCO) were grown in high glucose Dulbecco’s Modified Eagle Medium (DMEM, GIBCO) with 1X Penicillin-Streptomycin (Nacalai Tesque), 2 mM L-Glutamine (Biological Industries), and 10 % heat-inactivated FBS (GIBCO), at 37°C and 5% CO_2_.

### Antibody Production and Purification

Pertuzumab/Trastuzumab IgE VH family and Pertuzumab/Trastuzumab IgG1 VH/Vκ family variants were produced as previously described (30, 31). Pertuzumab and Trastuzumab VH5/VH3 IgG1 variants paired with different Vκ (Vκ1 to Vκ6) were produced in the same manner. A protein L column was used for IgE purification, while a protein G column was used for IgG1.

### Bio-Layer Interferometry Studies and Cross Metal Reactivity

Association, dissociation, and equilibrium dissociation rate constants of the Igs to the Ni-NTA were measured as was performed previously (30, 31) through direct binding of the antibodies to NTA biosensors (Fortebio) recharged with 10 mM NiCl2 for 1 min on the Octet Red96 system (Fortebio).

For cross metal reactivity measurements, Ni-NTA biosensors were stripped with low pH 1.7 10 mM glycine and recharged with 10 mM of Cobalt (II) Chloride (CoCl_2_), Nickel (II) Chloride (NiCl_2_), or Copper (II) Chloride (CuCl_2_), before binding with 100 nM of Pertuzumab VH5 IgE variants.

### Modeling Trastuzumab and Pertuzumab VH3, VH5, and VH7 IgE and VH5 IgG1

Models of Pertuzumab and Trastuzumab VH3, VH5, and VH7 IgEs paired with their respective Vκ1 were constructed previously (30). The Pertuzumab VH5-Vκ1 IgG1 model was built *via* displacement of the Cϵ of Pertuzumab VH5-Vκ1 IgE structure with Cγ (IgG1) followed by minimization and equilibration (1 ns, explicit solvent with sequential constant volume and pressure, NVT and NPT ensembles, respectively) using GROMACS 2019.3 (37). Displacement of Vκ1 with Vκ3 and Vκ6 of the Pertuzumab and Trastuzumab VH5 IgG1 gave rise to the other models.

### Molecular Dynamics (MD) Simulation of Trastuzumab and Pertuzumab IgE^Fab^ and IgG1^Fab^

To maintain the neutral charges and avoid interference at the two termini of each single Fab domain, the Fab models of whole Pertuzumab and Trastuzumab IgE and IgG1 variants were extracted and capped with ACE/NME at the N- and C-terminus respectively. The system was then relaxed by short minimization (5000 steps) and heated in gradual thermal baths from 0-100 K (with constant volume) and then from 100-300 K (with constant pressure). The system was then equilibrated (1 ns) and followed by 100 ns production applying explicit solvent model in triplicates (3×100 ns). The MD simulations were carried out with random velocities and constrained by the Langevin temperature equilibration scheme to thermalize the systems at 300K at time steps of 2 fs, using AMBER14 (38).

### Blind Docking of Nickel (Ni^2+^)-Bound NTA Onto the Pertuzumab and Trastuzumab VH3, VH5, and VH7 IgE^Fab^

The Ni-bound NTA (nitrilotriacetic acid) was first constructed using topologies of NTA (from PDB: 1GVC) and of Ni^2+^ ion (from PDB: 4PPT). AM1-BCC charge method (implemented in antechamber) was applied using Chimera (39) to compute partial charges of the constructed Ni-bound NTA, termed Ni-NTA.

Blind dockings using Autodock Vina (40) with Ni-NTA (as ligand) and the Pertuzumab and Trastuzumab VH3, VH5, or VH7 IgE Fab domains (Cϵ2 onwards were assumed to be insignificant, thus excluded) were performed. Ni-NTA internal bonds were kept rigid to minimize confounding binding to the respective Fabs while also constraining the flexibility of the free Ni^2+^ from binding to the deeply buried cavities. A 1-Å grid box was placed at the molecule center and extended to cover the whole Fab domain. Pre-defined parameters of Ni^2+^ ion in the AutodockTools packages were used. The dockings were performed for 10 replicates that represent 10 different IgE^Fab^ conformations of each variant, resulting with 10×1000 conformers for each and with exhaustive searching set at 128. A distance cut-off of 4 Å between the Ni^2+^ ion of the Ni-NTA and the interacting residue was used to determine the binding.

### Cryptic Pocket Detection

Using an online server CryptoSite (41), cryptic binding site detection was performed on the Fab domains of Pertuzumab and Trastuzumab VH3-Vκ1, VH5-Vκ1, or VH7-Vκ1 IgE variants. The predicted cryptic residues were then matched into identified pockets resulting from ‘mdpocket’ (42). Only internal (hidden) pockets or open channels (with isovalue ≥ 5) detected in more than 80% of the MD trajectories were selected and classified as dense (isovalue ≥ 8) or less dense (5 ≤ isovalue < 8) cavities. Pockets were deemed cryptic if they contained at least one cryptic residue predicted by the CryptoSite.

### Structural Analysis of Protein-Metal Complexes

Protein-metal complexes of Ni^2+^, Cu^2+^, and Co^2+^ from Protein Data Bank (PDB, accessed in June 2020) were enquired using the advance ligand search function with keywords: ‘NI’, ‘CU’, or ‘CO’ in the “Identifier” search. Only protein complexes containing standalone metal ligands were retrieved. The search was further narrowed to immune protein complexes by extending the keywords to “immune”, “antibody”, “MHC”, and “TCR”.

### Statistical Analysis

All KD (M), ka (1/Ms), and kd (1/s) measurements were performed in at least duplicates at concentrations from 200 nM to 3.125 nM of the antibodies using the Octet RED96 system and analysis software. Measurement responses of less than 0.1 nm for at least three concentrations were classified as immeasurable or “poor responses”.

## Results

### The Role of VH on Additional Ni-Binding

Recombinant Pertuzumab and Trastuzumab IgE and IgG variants (**Figure S1**) of VH families: VH1 to VH7 were paired with their respective Vκ1 light chains and analyzed with respect to nickel-attached nitrilotriacetic acid biosensor (Ni-NTA) binding using the Octet RED96 bio-layer interferometry system. Measurements using NTA alone (without Ni^2+^) with Pertuzumab VH5 IgE ruled out NTA as the interacting ligand (**Figure S2**), providing confidence that experiments measured only Ni interactions.

**Figure 1:**
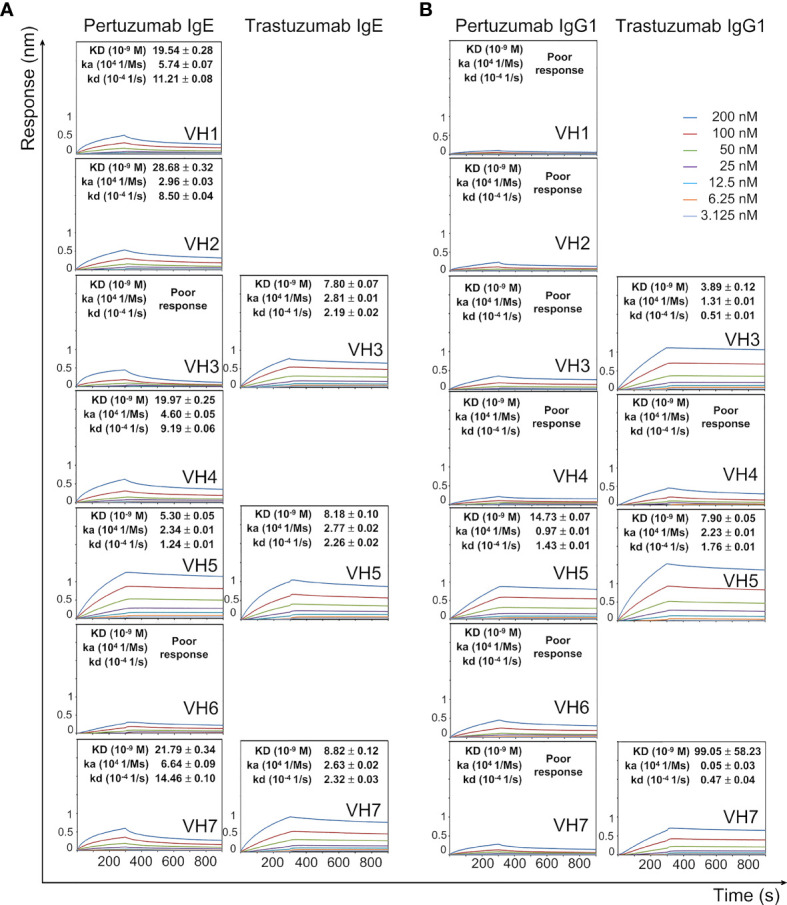
Dissociation equilibrium constants (KD) of Pertuzumab and Trastuzumab IgE **(A)** and IgG1 **(B)** of different VH families with the Ni-NTA biosensor. The measurements were performed at concentrations from 200 nM to 3.125 nM of the antibodies. Values of KD (M), ka (1/Ms), and kd (1/s) were determined using the Octet RED96 system. The X-axis depicts the time (in seconds) while the Y-axis depicts the binding responses (nm). All the experiments were conducted in at least duplicates. “Poor response” reflects measurement responses that are less than 0.1 nm for at least three concentrations. Variants that could not be produced are left empty.

All Pertuzumab IgE VHx variants (except for VH3 and VH6) interacted with the Ni-NTA sensor (determined by equilibrium dissociation constants KD), with the VH5 variant having the lowest KD (best binding, shown in **Figure 1A**). For Pertuzumab IgG1 variants, all but VH5 had poor responses in the measurements (**Figure 1B**), indicating that the swapping of heavy chain constant region Cϵ (IgE) to Cγ (of IgG1) ablated the Ni-NTA interactions. Conversely, the swap of Cϵ to Cγ improved Ni-NTA interactions for Trastuzumab VH3 and VH5 variants, thereby showing (e.g., in the Pertuzumab data) that the CH contributed to Ni-binding at the V-regions.

There were varying Ni-NTA KD measurements within the Pertuzumab and Trastuzumab VH variants (**Figure 1**). Using Pertuzumab CDRs, VH5 IgE and IgG1 had the best binding (KD ~5.30 nM and ~14.73 nM, respectively) among all the Pertuzumab variants. When grafted with Trastuzumab CDRs, both the VH3 and VH7 IgE and IgG1 variants as well as VH5 IgG1 yielded lower KD (better binding) while VH5 IgE showed a reverse trend. We could not test the other Trastuzumab VH variants as they could not be produced as previously mentioned (30).

### Ni^2+^ Binding Glutamines

While Ni^2+^ binding to histidine (H) is canon (43), the non-canonical glutamine (Q) could also bind Ni^2+^ as observed for HLA-DR52C (44) and in a single domain antibody VHH (45). Analysis of 8,054 Ni^2+^ interactions (0.4% of Q and 59.2% of H) from 1,810 protein-Ni (II) complexes (from PDB in June 2020) revealed that structural stretches of residues accounted for the Ni binding.

**Figure 2:**
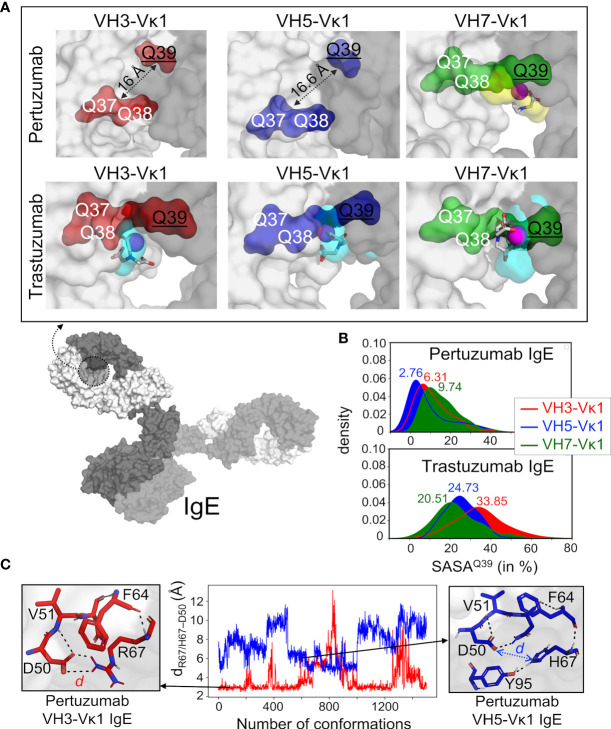
Structural analyses of glutamine from Pertuzumab and Trastuzumab VH3, VH5, and VH7 IgE variants in binding Ni-NTA. **(A)** Model of whole IgE (L-chain in white surface and H-chains in gray surface) reveals the formed pocket at the VHx–Vκ1 interface (dashed circle). The Ni-NTA bound region of the variants VH3 (red), VH5 (blue), and VH7 (green) shows the glutamine (Q) stretch constituted by the VH Q39 (underlined) and Vκ1 Q37-Q38 to form a stable cryptic pocket (capacity of which is shown in cyan transparent surface) in the Trastuzumab IgE variants or a transient non-cryptic pocket (yellow transparent surface) in the Pertuzumab VH7. The bound Ni-NTA is shown in magenta spheres and interacts at the pocket in both Pertuzumab and Trastuzumab. **(B)** Relative solvent accessible surface area (SASA) of the Pertuzumab and Trastuzumab VH Q39 residue in all the variants to demonstrate the deeply buried Q39 (SASA< 10%) of the Pertuzumab variants. **(C)** Distance *d* between residue 67 (R67 in VH3 or H67 in VH5) and H-chain CDR2 D50 of Pertuzumab VH3-Vκ1 IgE (red) and VH5-Vκ1 IgE (blue) during the simulation. Structural visualizations were generated using PyMOL 2.3.2 (46). An augmented reality (AR) presentation of the Ni-NTA binding to Trastuzumab VH5 IgE glutamine-forming pocket could be viewed using our ‘‘APD AR Holistic Review’’ app available on both Google and Apple app stores [for more details see ref (47, 48)].

The H and Q residue counts within both Pertuzumab and Trastuzumab V-regions showed no observable pattern (**Table S1**). Blind dockings with Ni-NTA of the VH3, VH5, and VH7 Pertuzumab and Trastuzumab IgE Fab regions (containing VHx-Vκ1, Cκ, and Cϵ1) showed interactions at the glutamine (Q) stretches (**Figure 2** and **Figure S3**). Specifically, Ni-NTA interacted with the Q-stretch involving residues Q38 (Vκ1 FWR2) and Q39 (VH3, VH5, and VH7 FWR2) of the Trastuzumab IgE variants (**Figure S3**) with Q37 of Vκ1 FWR2. This Q-stretch is found in a stable (3×100 ns simulation trajectories) cryptic pocket at the interface of the heavy and light chains (cyan in **Figure 2A**, see methods and **Table S2**) in Trastuzumab but not Pertuzumab IgE, Instead, a lower density cavity formed in Pertuzumab VH7 IgE by Q39 (VH7 FWR2), Q37 and Q38 of the Vκ1 (yellow in **Figure 2A**). Agreeing with experimental measurements where Pertuzumab VH7 IgE binds Ni-NTA poorer than Trastuzumab VH7 IgE (**Figure 1A**), a lower populated Ni-NTA cluster occurred at Q39 of the VH7 FWR2 as opposed to the corresponding area for Trastuzumab VH7-Vκ1 IgE (**Figure S3**).

The lack of Q-stretch pockets within Pertuzumab VH3-Vκ1 and VH5-Vκ1 IgEs were attributed to the distance between the buried VH Q39 residue from Vκ1 Q37 and Q38 (**Figures 2A, B**). Ni-interactions by Vκ1 Q89, VH5 H67 (in FWR3), and heavy chain CDR2 D50 in both VH3 and VH5 of Pertuzumab IgEs were also found (**Figure S3**). Interestingly, H67 of VH5-FWR3 is present in both Trastuzumab and Pertuzumab models suggesting its importance in the Ni engagement.

**Figure 3:**
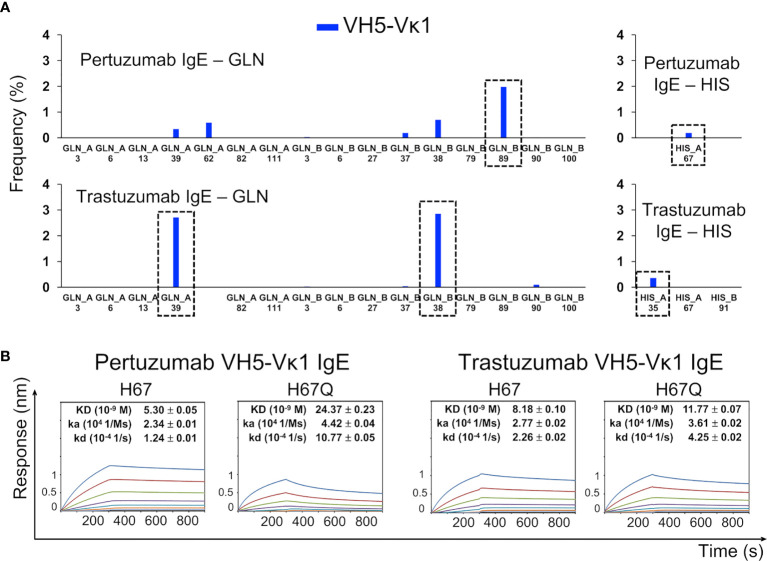
The Ni-NTA binding region of the Pertuzumab VH5–Vκ1 IgE. The main residue for the interaction is H67 of VH5 FWR3, whereas for Trastuzumab VH5–Vκ1 IgE, it is at the Q-stretch containing VH5 Q39 at FWR2. **(A)** Distribution of the Ni-NTA docked conformers showing all the glutamine (GLN) and histidine (HIS) present in the V-region of the Pertuzumab and Trastuzumab VH5–Vκ1 IgE variants. The predominant binding residue locations are highlighted in dashed boxes. **(B)** KD of the Pertuzumab and Trastuzumab VH5–Vκ1 IgE variants including H67Q mutation.

Site-directed H67Q mutagenesis (in VH5-10 and other VH5 germlines) of both Pertuzumab and Trastuzumab VH5 IgEs disrupted the Ni binding, having pronounced effects in Pertuzumab VH5^H67Q^ IgE (~5-fold KD increase) and less in Trastuzumab VH5^H67Q^ IgE (~1.5-fold KD increase, **Figure 3**). Although H67 is present in the VH5 FWR3 of both antibodies, it is only in Pertuzumab VH5 that the residue synergized with Vκ1 Q89 for Ni-binding. This was contrary to the Q39-Q37/Q38 stretch in Trastuzumab VH5 that played the key role of Ni engagement (**Figure 3**).

The poor responses in Pertuzumab VH3 IgE Ni engagement (**Figure 1A**) agreed with the blind docking results showing general scattering with denser congregation around D50 of the VH CDR2 (**Figure S3**). This D50 formed a polar contact network extending to residue 67 of the VH FWR3, i.e., R67 and H67 of Pertuzumab VH3 and VH5 IgE, respectively (**Figure 2C**). When stable, the D50-R67 interaction decreased Ni-NTA interaction whereas its absence in Pertuzumab VH5 IgE permitted greater Ni-NTA accessibility.

### The Role of Vκ in Ni-Binding

To study the role of Vκ, Pertuzumab and Trastuzumab Vκ1 to Vκ6 light chains were paired with their respective VH3 and VH5 (lowest KD in Ni-NTA binding in **Figure 1B**) IgG1 variants.

**Figure 4:**
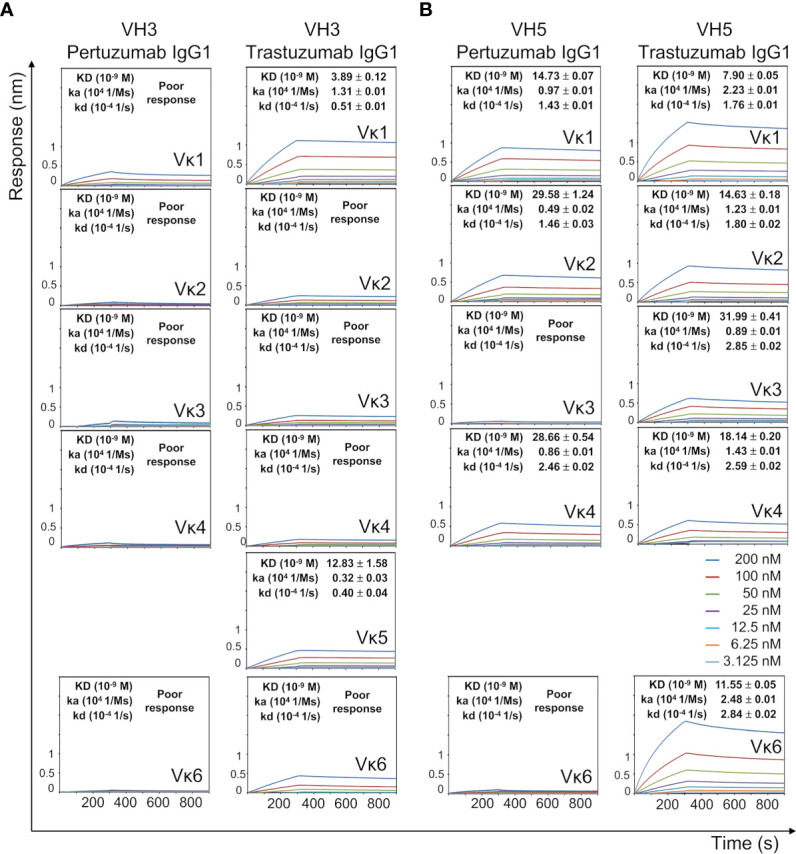
Ni-NTA dissociation equilibrium constants (KD) of Pertuzumab and Trastuzumab VH3 **(A)** and VH5 **(B)** IgG1 variants paired with different Vκs. The experiments were performed at concentrations from 200 nM to 3.125 nM of the antibodies. Values of KD (M), ka (1/Ms), and kd (1/s) were determined using the Octet RED96 system. The X-axis depicts the time (in seconds) while the Y-axis depicts the binding responses (nm). All the experiments were conducted in at least duplicates. “Poor response” reflects measurement responses that are less than 0.1 nm for at least three concentrations. Variants that could not be produced are left empty.

With the exception of Vκ5 that was not produced, all Pertuzumab VH3 IgG1 variants of the various Vκs showed poor Ni-NTA responses (**Figure 4A**). When Vκ1 was replaced with Vκ2, Vκ3, Vκ4, or Vκ6 for the Trastuzumab VH3 IgG1 variants, the Ni-NTA bindings could not be measured, although a higher KD was found with Vκ5 (increase from 3.89 nM to ~12.83 nM).

The VH5 IgG1 variants of both Pertuzumab and Trastuzumab had stronger Ni engagement than their VH3 counterparts (**Figure 4B**). This was especially so for Trastuzumab VH5 IgG1 when paired with Vκ2, Vκ3, Vκ4, and Vκ6 FWRs. Of the VH5 variants (except for the unproduced Vκ5), Pertuzumab IgG1s that were paired with Vκ3 and Vκ6 could not be measured (**Figure 4B**). Since sequence analysis showed only differences in the heavy/light CDRs between Pertuzumab and Trastuzumab VH5 IgG1, stronger Trastuzumab interaction is attributed to the CDRs. As Pertuzumab VH5-Vκ1 and VH5-Vκ3 IgG1 differed only at the light chain FWRs, the reverse binding trend demonstrated the synergistic contribution of both FWRs and CDRs of both heavy and light chains.

**Figure 5:**
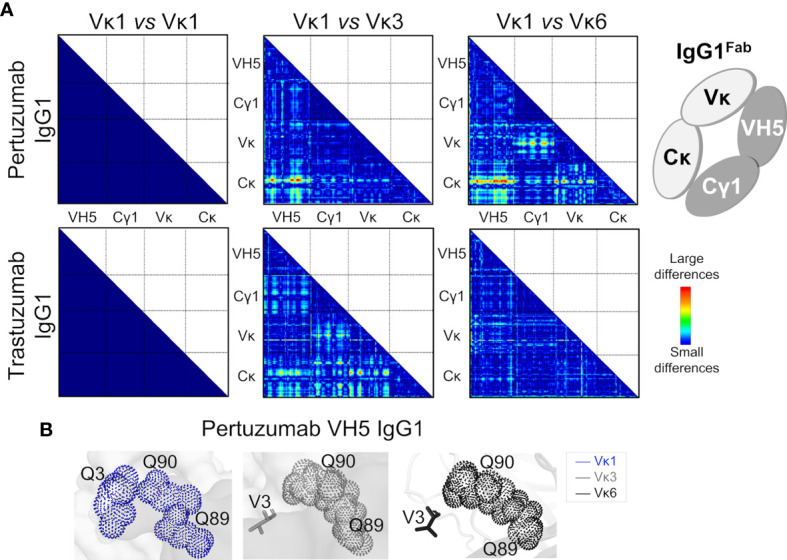
Effects of the different VH5-Vκ pairings in the Pertuzumab and Trastuzumab IgG1 variants. **(A)** Distance-based difference maps [generated using CMView (49)] between Fab domains of Pertuzumab and Trastuzumab VH5-Vκ3 and VH5-Vκ6 IgG1 variants (superimposed against the respective VH5-Vκ1 IgG1 as the reference) indicates more disruption by the Vκ3 or Vκ6 for Pertuzumab VH5 IgG1 variants, leading to varying changes in the heavy and light chain pairing. **(B)** An alternative Ni-NTA binding region in Pertuzumab VH5-Vκ1 IgG1 formed a Q-stretch of Vκ1 Q3, Q89, and Q90. These Q-stretches were incomplete due to the Vκ mutation Q3V in the Pertuzumab VH5-Vκ3 and VH5-Vκ6 IgG1 variants.

Analysis of the Q and H residue counts between the Vκ family variants (**Table S1**) showed relative consistency for both Pertuzumab and Trastuzumab VH5 IgG1 variants. Distance-based maps of VH5-Vκ1, VH5-Vκ3, and VH5-Vκ6 Pertuzumab and Trastuzumab IgG1^Fab^ variants showed that Vκ changes caused conformational changes while retaining VH5, Cγ1, and Cκ. The displacement of Vκ1 with Vκ3 or Vκ6 more pronouncedly disrupted Pertuzumab VH5 IgG1 variants (red color in **Figure 5A**) with respect to the Cκ accommodation. Displacement to Vκ6 shifted Vκ6 from Cκ and Cγ1 of the Pertuzumab IgG1 variants. In comparison, smaller effects were observed for Trastuzumab VH5-Vκ3 and VH5-Vκ6 IgG1 (blue and green in **Figure 5A**).

Blind dockings of Ni-NTA to the Trastuzumab VH5-Vκ1, VH5-Vκ3, and VH5-Vκ6 IgG1 Fab revealed Ni-NTA interactions at the Q-stretch of VH5 Q39 and Vκ Q37-Q38 of the heavy-light chain interface, as also observed for Trastuzumab VH5-Vκ1 IgE (**Figures S3** and **S4**). Given that the heavy and light chain geometry of the Trastuzumab VH5 IgG1^Fab^ were not significantly perturbed when swapping the Vκ, the Ni-NTA interactions remained consistent with the KD measurements (**Figure 4**).

Interactions of Ni-NTA with Vκ1 Q89 in the Pertuzumab VH5-Vκ1 IgG1 were found diminished in Pertuzumab VH5-Vκ3 IgG1 and abolished in the Pertuzumab VH5-Vκ6 IgG1 (**Figure S4**). Interestingly, Vκ1 Q89 formed another Q-stretch with the adjacent Vκ1-CDR3 Q90 and the distant Vκ1-FWR1 Q3. However, this Q-stretch is incomplete in the VH5-Vκ3 and VH5-Vκ6 Pertuzumab IgG1 variants due to the Q3V mutation in both Vκ3 FWR1 and Vκ6 FWR1 (**Figure 5B**), compromising the Ni-NTA interaction (**Figure 4B**). As in the Trastuzumab VH5-Vκ1 IgE, residue H67 was not involved in all three Trastuzumab VH5-Vκs IgG1 variants.

### Binding of Metal Ions

**Figure 6:**
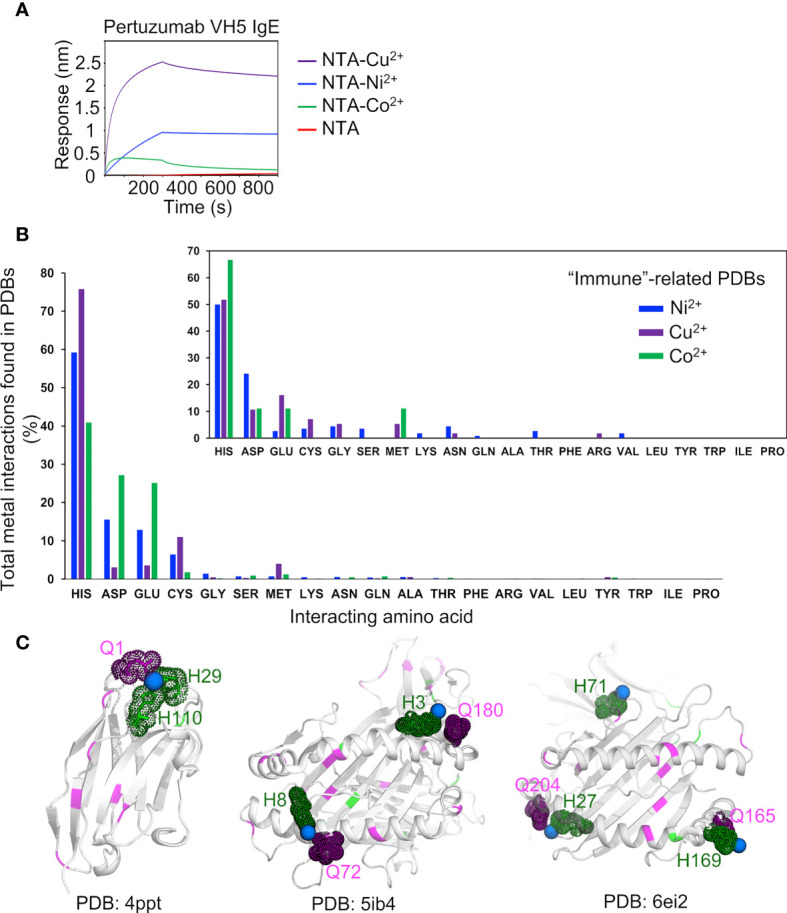
Analysis of metal binding ability. **(A)** Binding responses of the Pertuzumab VH5 IgE variant to different metal ions (Ni^2+^, Cu^2+^, and Co^2+^) recharged on the NTA sensors. **(B)** Numbers of the metal interactions in the retrieved protein-metal complexes from Protein Data Bank. The smaller offset plot showed the metal interaction results of a subset of immune-related protein complexes found by using independent keywords: “immune”, “antibody”, “MHC”, and “TCR”. **(C)** Structural presentation of a VHH antibody complexed with Ni^2+^ (PDB: 4PPT), HLA-B*27:05 (PDB: 5IB4), and HLA-A68 (PDB: 6EI2) complexed with a peptide and Ni^2+^, where glutamines (magenta) are found to be involved in (e.g., in 4PPT) or closed to the Ni^2+^ binding region with histidine (green). The Ni^2+^ ions are represented by blue spheres.

Measuring the interactions with various metal ions Cu^2+^ and Co^2+^ recharged onto the NTA sensors, Pertuzumab VH5-Vκ1 IgE bound the other metals in the order of Cu^2+^ > Ni^2+^ > Co^2+^ (**Figure 6A**) as per Irving-Williams’ order (50). From the analysis of protein-metal complexes (1,409 for Cu^2+^; 1,810 for Ni^2+^; and 716 for Co^2+^ in PDB, accessed in June 2020), histidine expectedly bound multiple metals, followed by aspartate, glutamate, cysteine, methionine, serine, and glutamine (**Figure 6B**). Only a few of these complexes are immune-related (12, 26, and 14 complexes with the metal ions, **Figure 6B**, small offset) and histidine was shown as the main residue interacting with all the three metal ions while glutamine bound only Ni^2+^. Notably, three protein-Ni^2+^ complexes (a single VHH antibody domain, PDB: 4PPT, and two HLA-peptide complexes, PDB: 5IB4 and 6EI2, shown in **Figure 6C**) contained both histidine and glutamine in their Ni^2+^ binding regions where glutamine either directly weakly interacted (4PPT) or was within close proximity to the Ni^2+^ binding histidine (5IB4 and 6EI2).

## Discussion

Through unsuccessful attempts to measure IgE interaction with His-tagged FcϵRIα *via* protein L immobilization (**Figure S5**) thereby eventually utilizing Ni-NTA biosensors, we found that Pertuzumab VH5 IgE interacted uniquely with Ni-NTA immobilized FcϵRIα-His (30). The conclusion of VH influencing Fc-FcϵRIα engagement of the work remained valid due to (i) the similar KD of Trastuzumab VH3, VH5, and VH7 IgE variants to Ni-NTA (**Figure 1A**) but with varying KDs to FcϵRIα-His (30), and (ii) the lack of Ni effects on Pertuzumab VH6 IgE binding Ni-NTA FcϵRIα-His (present study). With respect to VH effects on the FcR engagement, we show here that the VH of IgEs can also engage Ni without compromising antigen recognition.

Systematic displacement of the FWRs and CDRs in Pertuzumab and Trastuzumab IgE and IgG1 revealed contribution of these regions to Ni^2+^ engagement. Trastuzumab variants (except for VH5-Vκ1 IgE) bound better to the Ni-NTA when compared to the Pertuzumab IgE and IgG1. Further experiments with other heavy metals and NTA alone showed the interaction with Ni to be from Q-stretches. Since Trastuzumab and Pertuzumab are clinical therapeutics, the holistic (27) analysis of antibodies here clearly caution for sagacious design of biologics (51) to avoid possible interactions that may cause side effects.

Swapping the Cϵ (IgE) to Cγ (IgG1) failed to restore the Ni^2+^ binding (completely abolished in Pertuzumab VH3 IgE) demonstrating the distal effects of the CH. Despite possessing the known metal-binding histidine cluster “HEALHNH” (43) in Cγ, there was no notable Ni engagement in most of the Pertuzumab VHs IgG1 variants, consistent with previous findings (17).

Of the IMGT VH/Vκ families and germlines sequences, only VH3, VH4, Vκ1 (**Tables S3** and **S4**), and VH5 frameworks had an extra histidine, yet this is only a portion of the overall antibody population (52). Since IgE is in lower quantities than IgGs in blood (53), nickel binding IgEs due to the extra histidine is of very small odds.

The modulation of Ni-binding by differential Vκ pairing while retaining histidine content showed the contribution of the V- and C- regions of the IgE variants in the structural formation of Ni- binding Q-stretches (continuous clusters of at least 3 glutamines). These Q-stretches occurred at the heavy and light chain interface of the Trastuzumab IgE variants or at scaffolds between Vκ1 FWR1 and CDR3 of the Pertuzumab VH5 IgG1 variants. Since many of our IgE variants exhibited better Ni binding than their IgG1 counterparts, the phenomenon is more prevalent in IgEs than IgGs.

Hapten-specific T cells (2) showed that Ni^2+^ bound MHC-peptide complexes have Ni^2+^ bridges between the histidine on the HLA-DR β-chain and the TCR α chain hypervariable region (14). Notwithstanding the Ni^2+^ engagement of the rheumatoid associated (15) peptide-bound HLA-B*27:05 complex (PDB: 5IB4), we did not find other evidence. This may be because the reported peptide underwent an Ni^2+^ induced reorientation *via* an energetically driven mechanism congruent to the Ni^2+^ binding of the H67 in Pertuzumab but not Trastuzumab VH5-Vκ1 IgE.

While metal binding can influence antigen presentation of both MHC class I and II (15, 44), the atypical non-antigenic mechanism of Ni^2+^ here can directly activate both TCR in Type IV or cross-link IgE on mast cells to trigger Type I (11) hypersensitivities. While linked recognition may occur in patients with dual clinical manifestations (4), it is extremely rare. Nonetheless, the mechanism in the IgEs was shown to be of non-conventional antigenic binding and more like that of antibody superantigens proteins A and L to be not-involving CDRs alone. Thus we propose the inclusion of nickel as new dual superantigen-like molecule if not a superantigen.

We note that the classification of superantigen and superantigen-like molecules to be very contentions with some in the immunology recognizing T-cell superantigens and not that of antibodies, and that nickel was excluded as a TCR superantigen (14, 35), However, molecular mechanisms that we uncover here may underlie why “specific combination of α- and β-chains” are required for Ni-binding by TCRs (35) to form the Ni-binding amino acid stretches required as in our IgEs here.

Although we are unable to clinically validate the potential of the mechanism here underlying the cause of type I and IV nickel allergies given that access to a rare patient co-manifesting both nickel allergies is challenging, much less finding the right T- and B-cell clones in the patient, we have demonstrated that the phenomenon is possible with clinical therapeutics. The mechanism can underlie the metal-protein interaction reported in the two allergy types (11, 54) where natural VDJ generated antibodies and therapeutic antibodies could possess unintended Q-stretches. Such unintended nickel binding by antibodies can go undetected unless specifically looked for given the uncompromised intended antigen interaction (in our case Her2). Such a mechanism can underlie the dermatological side effects (19–21) associated with some biologics therapy and why prolonged exposure to nickel can elicit type I and IV hypersensitivities in later life akin to allergic rhinitis and *Staphylococcus aureus* colonization *via* protein A (55–58). Given that nickel is ubiquitous in modern day living (1), the selection of such IgEs or even T-cells is certainly a high possibility.

With previous evidence that some VH5 IgEs can exhibit prolonged interactions with FcϵRIα (30), thereby likely becoming the more dominant VH family found in asthmatics (59), the ability to bind to nickel in our VH5 IgEs seem to dovetail the rise of the otherwise rare nickel Type I allergy. Together, these findings demonstrate the importance of VH5 biased IgEs to which the actual discovery in allergic patients is necessary. The rarity of this may be attributed to the low percentage of IgE to total Ig, that together with the lower usage of VH5 compared to the other VH families of 1, 3, 4, and 6 (60), explain why type I nickel allergies are not more prevalent. However, given that the phenomenon can occur with VH3-IgEs, it is prudent to minimize unnecessary exposure to nickel and other metal as with that of other superantigens.

As the binding of Ni occurs at the V-regions, the FcR engagements are unlikely to be affected. Thus, the IgG counterpart may function to mop up Ni-protein complexes that may otherwise cross-link sensitized mast cells or moderate the activation of dendritic cells/keratinocytes for detoxification (61). Further studies of Ni-specific antibody isotype levels in patients would be necessary to investigate this.

Since Ni can act as adjuvants to other metals (62), there are possible exploitation of the mechanism to engineer better antibodies similar to the Bi-specific T-cell engagers (BiTEs) technology where nickel binding may attract associated TCRs. Further tailoring Ni towards such uses is required, with mindfulness of inducing Ni hypersensitivity and its effects.

In conclusion from our systemic displacement of antibody regions, Ni-binding antibodies were found to do so *via* a combination of factors involving the CDRs and FWRs of both VH and Vκ chains, and the CH to form inducible structural Q-stretches without losing antigen specificity. This superantigen-like property can be exploited in potential therapeutic interventions as well as in biotechnological purification and antibody engineering purposes.

## Data Availability Statement

The original contributions presented in the study are included in the article/**Supplementary Material**. Further inquiries can be directed to the corresponding author.

## Author Contributions

WHL, WLL, and JJP produced the recombinant antibodies and performed the biolayer interferometry experiments. JYY performed the framework germline experiment. CTTS performed the computational simulation and analyses. CTTS, WHL, and SKEG analysed the results and wrote the manuscript. SKEG designed and supervised all aspect of the study. All authors contributed to the article and approved the submitted version.

## Funding

The work was supported by A*STAR core funds.

## Conflict of Interest

SKEG was employed by APD SKEG Pte Ltd., Singapore, Singapore.

The remaining authors declare that the research was conducted in the absence of any commercial or financial relationships that could be construed as a potential conflict of interest.
