# Supplementary material for: Molecular Insights of Nickel Binding to Therapeutic Antibodies as a Possible New Antibody Superantigen
Source: Front Immunol. 2021 Jul 8;12:676048. doi: 10.3389/fimmu.2021.676048 (PMC8296638; doi:10.3389/fimmu.2021.676048)
Supplement: Supplementary file 1 [file DataSheet_1.docx]

Supplementary Material

# Supplementary Figures

**
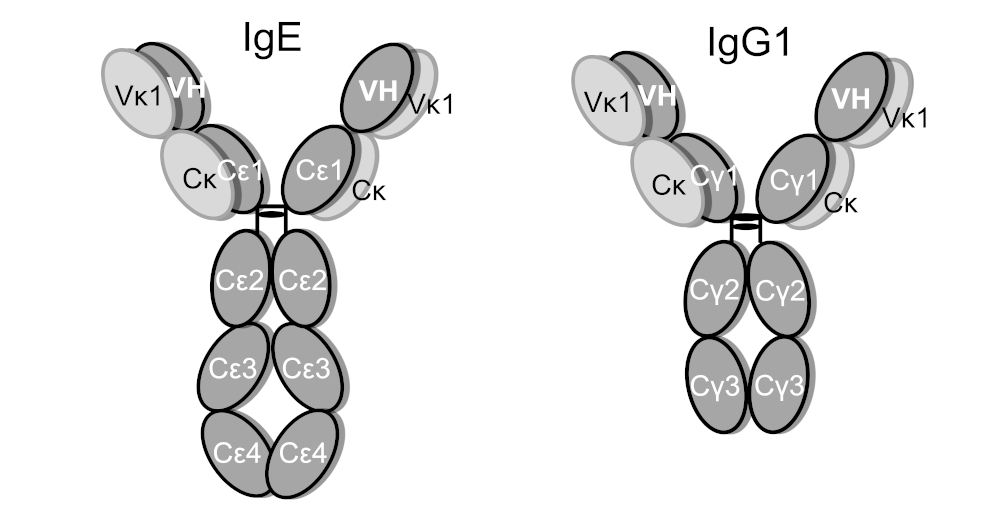
**

**Supplementary Figure S1.** Schematic of full-length IgE and IgG1 antibodies showing pairing of heavy and light chain regions.

**
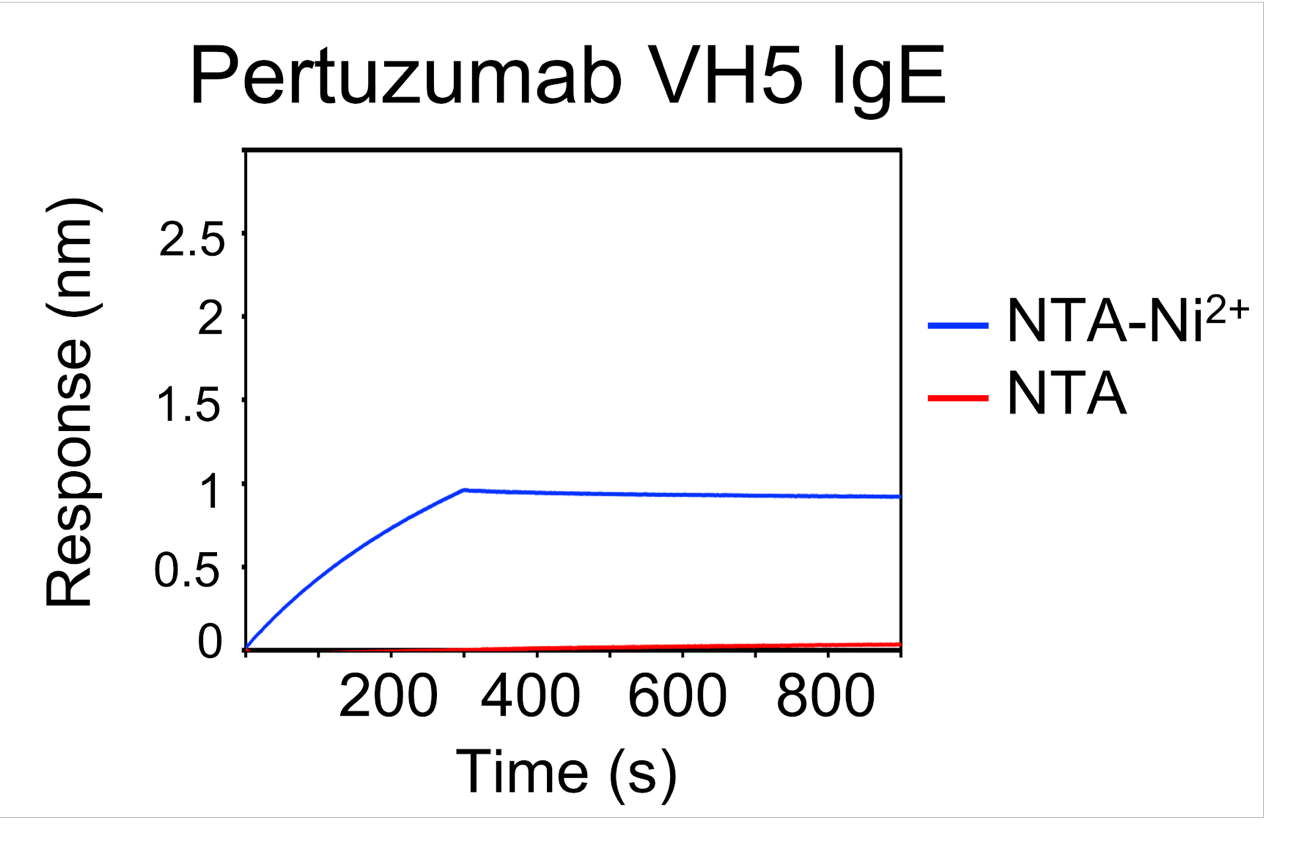
**

**Supplementary Figure S2.** Binding response of the Pertuzumab VH5 IgE to the Ni-NTA sensor that was recharged with NiCl_2_ (NTA-Ni^2+^) versus NTA with only water (NTA only) to rule out interactions with the NTA sensor. The result shown here is a part of those presented in Fig. 6A.

**
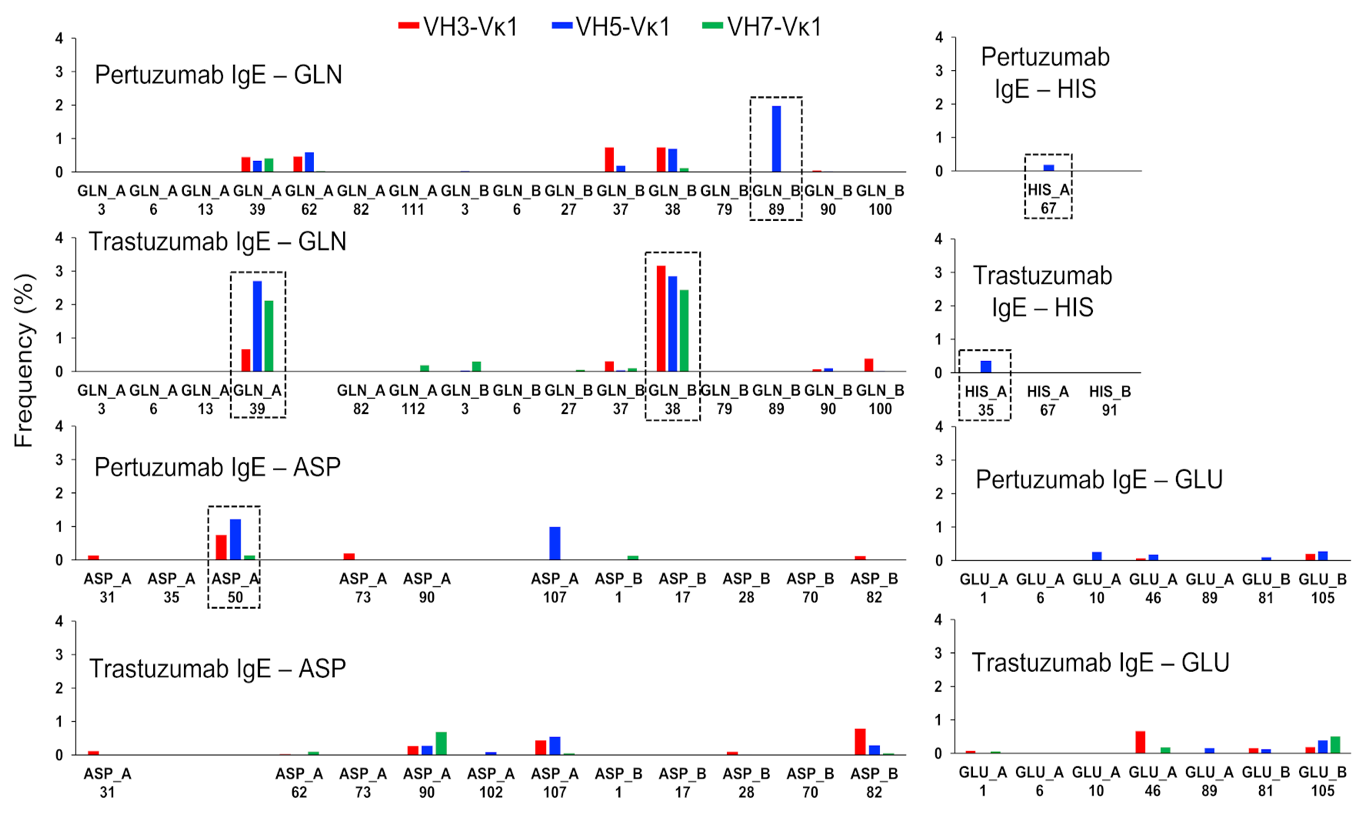
**

**Supplementary Figure S3.** Distribution of the Ni-NTA conformers clustered at the residues of interest such as glutamine (GLN), histidine (HIS), aspartate (ASP), and glutamate (GLU) on the Pertuzumab and Trastuzumab IgE V-regions of VH3, VH5, and VH7 variants. Measurements were not possible at cysteine residues. Predominant clusters of the Ni-NTA conformers are highlighted in dashed boxes. Residues in the heavy and light chains are labeled as chains A and B, respectively.

**
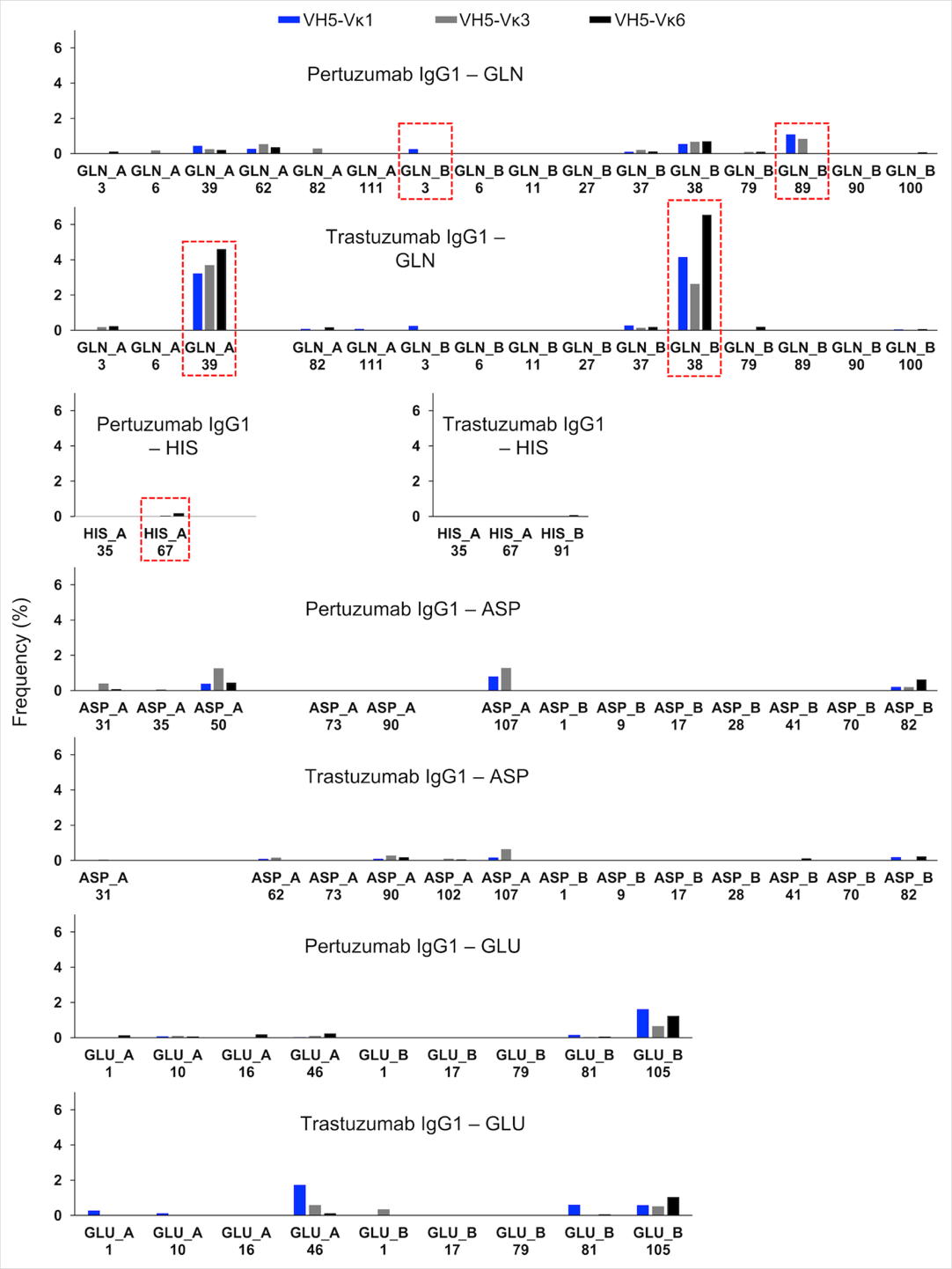
**

**Supplementary Figure S4.** Distribution of the Ni-NTA conformers clustered at the residues glutamine (GLN), histidine (HIS), aspartate (ASP), and glutamate (GLU) on the Pertuzumab and Trastuzumab IgE V-regions of VH5 paired with Vκ1, Vκ3, and Vκ6. The clusters of interest of the Ni-NTA conformers are highlighted in dashed boxes. Residues in the heavy and light chains are labeled as chains A and B, respectively.

**
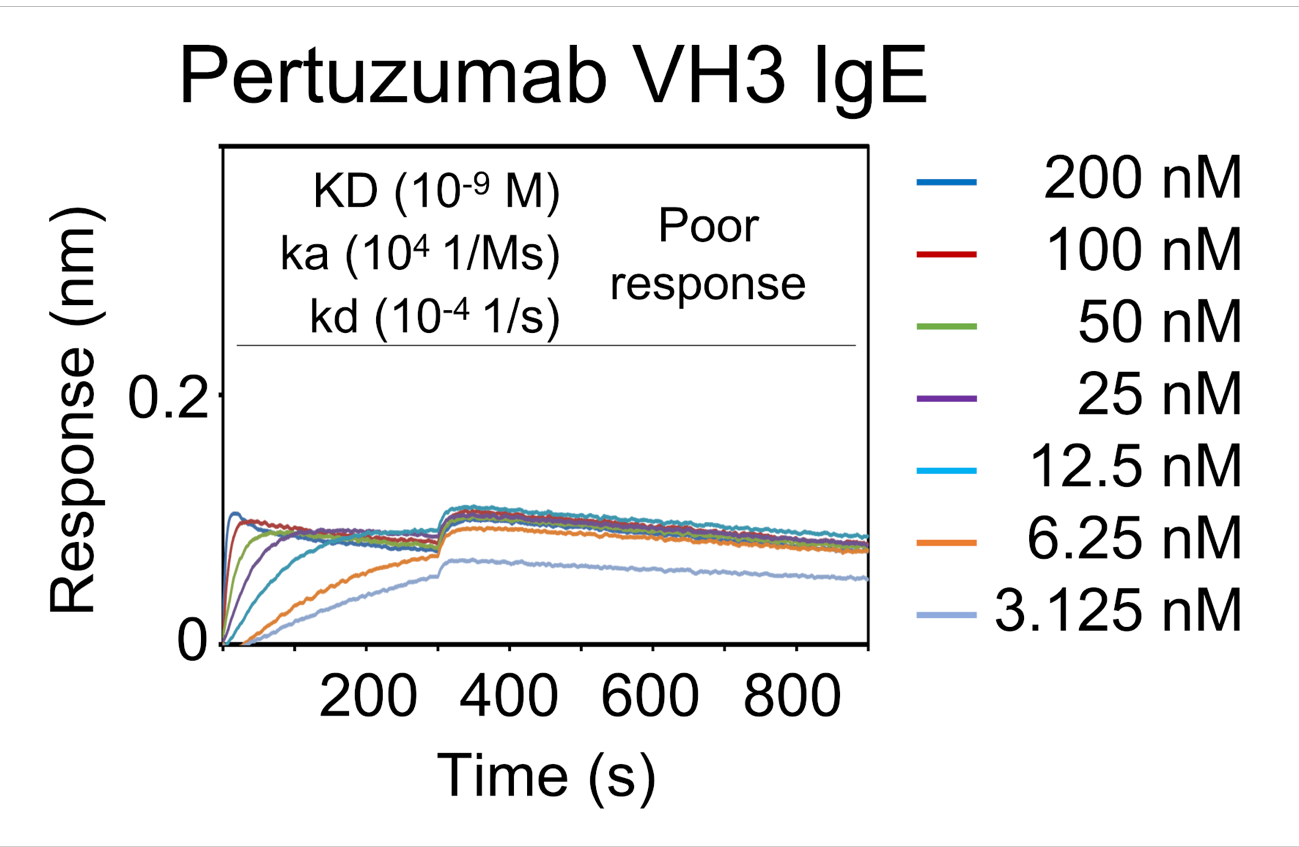
**

**Supplementary Figure S5.** Dissociation equilibrium constants (KD) of the FcεRIα to the immobilized Pertuzumab VH3 IgE (using Protein L biosensor). Biolayer interferometry measurements of the FcεRIα at various concentrations from 200nM to 3.125nM. Values of KD (M), ka (1/Ms), and kd (1/s) were measured and calculated using the Octet RED96 system. The X-axis depicts the time (in seconds) and the Y-axis depicts the binding response (nm).

# Supplementary Tables

**Supplementary Table S1.** Histidine, Glutamine, Glutamate, Aspartate, and Cysteine residues in the antibody variants of the study. Where the variants share the same domain, only one value is presented for that domain count. For examples, all Pertuzumab (or Trastuzumab) VHs (or Vκs) variants share the same heavy H-chain (or light L-chain) CDRs, respectively. All IgE (or IgG1) variants share the same constant regions CHs. Cκ is the only L-chain constant region in this study.

|  |  | **Pertuzumab / Trastuzumab** | | | | | | | | | | | | | |
| --- | --- | --- | --- | --- | --- | --- | --- | --- | --- | --- | --- | --- | --- | --- | --- |
|  |  | **Heavy Chain** | | | | | | |  | **Light Chain** | | | | | |
|  |  | **VH1** | **VH2** | **VH3** | **VH4** | **VH5** | **VH6** | **VH7** |  | **Vκ1** | **Vκ2** | **Vκ3** | **Vκ4** | **Vκ5** | **Vκ6** |
| **HIS (H)** | H-FWRs | 0 / 0 | 0 / 0 | 0 / 0 | 0 / 0 | 2 / 2 | 0 / 0 | 0 / 0 | L-FWRs | 0 / 0 | 0 / 0 | 0 / 0 | 0 / 0 | 0 / 0 | 0 / 0 |
|  | H-CDRs | 0 / 2 | | | | | | | L-CDRs | 0 / 2 | | | | | |
|  | CHs | 22 (IgE) – 18 (IgG1) | | | | | | | Cκ | 4 | | | | | |
| **GLN (Q)** | H-FWRs | 16 /14 | 12 /10 | 12 /10 | 18 /16 | 12 /10 | 20 /18 | 12 /10 | L-FWRs | 12 /12 | 10 /10 | 10 /10 | 12 /12 | 10 /10 | 12 /12 |
|  | H-CDRs | 2 / 0 | | | | | | | L-CDRs | 6 / 6 | | | | | |
|  | CHs | 38 (IgE) – 22 (IgG1) | | | | | | | Cκ | 12 | | | | | |
| **GLU (E)** | H-FWRs | 6 / 6 | 4 / 4 | 8 / 8 | 2 / 2 | 8 / 8 | 4 / 4 | 8 / 8 | L-FWRs | 4 / 4 | 8 / 8 | 10 /10 | 6 / 6 | 10 /10 | 10 /10 |
|  | H-CDRs |  |  |  | 0 / 0 |  |  |  | L-CDRs | 0 / 0 | | | | | |
|  | CHs | 38 (IgE) – 34 (IgG1) | | | | | | | Cκ | 14 | | | | | |
| **ASP (D)** | H-FWRs | 8 / 6 | 8 / 6 | 6 / 4 | 6 / 4 | 6 / 4 | 6 / 4 | 6 / 4 | L-FWRs | 8 / 8 | 8 / 8 | 4 / 4 | 10 /10 | 6 / 6 | 8 / 8 |
|  | H-CDRs |  |  |  | 8 / 8 |  |  |  | L-CDRs | 2 / 2 | | | | | |
|  | CHs | 36 (IgE) – 26 (IgG1) | | | | | | | Cκ | 10 | | | | | |
| **CYS (C)** | H-FWRs | 4 / 4 | 4 / 4 | 4 / 4 | 4 / 4 | 4 / 4 | 4 / 4 | 4 / 4 | L-FWRs | 4 / 4 | 4 / 4 | 4 / 4 | 4 / 4 | 4 / 4 | 4 / 4 |
|  | H-CDRs |  |  |  | 0 / 0 |  |  |  | L-CDRs | 0 / 0 | | | | | |
|  | CHs | 26 (IgE) – 18 (IgG1) | | | | | | | Cκ | 6 | | | | | |

**Supplementary Table S2.** Top predicted cryptic residues of the Trastuzumab VH3-Vκ1, VH5-Vκ1, and VH7-Vκ1 IgE using CryptoSite (Cimermancic *et al.*, 2016). The different cutoff scores between two consecutive residue results was set to 0.05 to determine the selected residues from the first highest score. The Q39 residues in the identified internal pockets are in bold.

| Variants* | Heavy chain | | | Light chain | | | CryptoSite score  (in descending order) |
| --- | --- | --- | --- | --- | --- | --- | --- |
| VH3-Vκ1 |  |  |  | PHE | B | 98 | 0.3578407 |
|  |  |  |  | PRO | B | 120 | 0.3373744 |
|  | TRP | A | 110 |  |  |  | 0.331121 |
|  | **GLN** | **A** | **39** |  |  |  | **0.3137926** |
| VH5-Vκ1 | **GLN** | **A** | **39** |  |  |  | **0.31053** |
|  |  |  |  | THR | B | 180 | 0.3089352 |
|  | ARG | A | 133 |  |  |  | 0.2860081 |
|  | THR | A | 152 |  |  |  | 0.2848823 |
|  |  |  |  | SER | B | 131 | 0.2757533 |
| VH7-Vκ1 | **GLN** | **A** | **39** |  |  |  | **0.3666808** |
|  |  |  |  | PHE | B | 98 | 0.350589 |
|  |  |  |  | THR | B | 180 | 0.3410676 |
|  | TRP | A | 47 |  |  |  | 0.3155691 |
|  |  |  |  | LYS | B | 39 | 0.2914044 |

*None of cryptic residues predicted for the Pertuzumab VH7-Vκ1 IgE were found to be involved in the identified internal pockets in the variant. The Q39 residue of this variant was not a predicted cryptic residue.

**Supplementary Table S3:** Antibody framework repertoire analysis. Analysis of different L-chain FWRs for presence of histidine and the estimated germline occurrences.

| Families / Germline | Extra histidine | Frequency* (%) |
| --- | --- | --- |
| Vκ1-5 | - | 11 |
| Vκ1-6 | - | >1 |
| Vκ1-8 | - | 2 |
| Vκ1D-8 | - | >1 |
| Vκ1-9 | - | >1 |
| Vκ1-12 | - | >1 |
| Vκ1-13 | - | >1 |
| Vκ1D-13 | - | >1 |
| Vκ1-16 | - | >1 |
| Vκ1-17 | + | >1 |
| Vκ1-27 | - | 2 |
| Vκ1-33 | - | 2 |
| Vκ1D-33 | - | 2 |
| Vκ1-39 | - | 7 |
| Vκ1D-39 | - | 7 |
| Vκ1D-43 | - | >1 |
| Vκ2 – ALL | - | 11 |
| Vκ3 – ALL | - | 36 |
| Vκ4 – ALL | - | 8.5 |
| Vκ5 – ALL | - | >1 |
| Vκ6 – ALL | - | >1 |

*reported by Tiller *et al.* (Tiller *et al.*, 2013).

**Supplementary Table S4:** Antibody framework repertoire analysis. Analysis of the different H-chain FWRs with respect to the presence of histidine and estimated germline occurrences.

| Families / Germline | Extra histidine | Frequency* (%) |
| --- | --- | --- |
| VH1 – ALL | - | 18 |
| VH2 – ALL | - | >1 |
| VH3-7 | - | 4.5 |
| VH3-9 | - | 2 |
| VH3-11 | - | 2 |
| VH3-13 | - | >1 |
| VH3-15 | - | 3 |
| VH3-20 | + | >1 |
| VH3-21 | - | 3.5 |
| VH3-23 | - | 11 |
| VH3-30 | - | 2 |
| VH3-33 | - | 2 |
| VH3-43 | - | >1 |
| VH3-48 | - | 4 |
| VH3-49 | - | >1 |
| VH3-53 | - | 2 |
| VH3-64 | - | >1 |
| VH3-66 | - | >1 |
| VH3-72 | - | >1 |
| VH3-73 | - | >1 |
| VH3-74 | - | >1 |
| VH4-4 | + | 2.5 |
| VH4-28 | - | >1 |
| VH4-30 | + | >1 |
| VH4-31 | - | 3 |
| VH4-34 | + | 7 |
| VH4-38 | + | N.A |
| VH4-39 | + | 7.5 |
| VH4-59 | - | 5.5 |
| VH4-61 | + | >1 |
| VH5-10 | + | N.A |
| VH5-51 | + | 5 |
| VH6 – ALL | - | >1 |
| VH7 – ALL | - | >1 |

*reported by Tiller *et al.* (Tiller *et al.*, 2013).
